# The relationship between sleep and behavior in autism spectrum disorder (ASD): a review

**DOI:** 10.1186/1866-1955-6-44

**Published:** 2014-12-11

**Authors:** Simonne Cohen, Russell Conduit, Steven W Lockley, Shantha MW Rajaratnam, Kim M Cornish

**Affiliations:** School of Psychological Sciences, Faculty of Biomedical and Psychological Sciences, Monash University, Melbourne, Australia; School of Health Sciences, Royal Melbourne Institute of Technology, Melbourne, Australia; Division of Sleep and Circadian Disorders, Brigham and Women’s Hospital, Boston, USA; Division of Sleep Medicine, Harvard Medical School, Boston, USA

**Keywords:** Autism spectrum disorder, Low-functioning autism, Sleep difficulties in ASD, Treating sleep in ASD

## Abstract

Although there is evidence that significant sleep problems are common in children with autism spectrum disorder (ASD) and that poor sleep exacerbates problematic daytime behavior, such relationships have received very little attention in both research and clinical practice. Treatment guidelines to help manage challenging behaviors in ASD fail to mention sleep at all, or they present a very limited account. Moreover, limited attention is given to children with low-functioning autism, those individuals who often experience the most severe sleep disruption and behavioral problems. This paper describes the nature of sleep difficulties in ASD and highlights the complexities of sleep disruption in individuals with low-functioning autism. It is proposed that profiling ASD children based on the nature of their sleep disruption might help to understand symptom and behavioral profiles (or vice versa) and therefore lead to better-targeted interventions. This paper concludes with a discussion of the limitations of current knowledge and proposes areas that are important for future research. Treating disordered sleep in ASD has great potential to improve daytime behavior and family functioning in this vulnerable population.

## Review

Autism spectrum disorder (ASD) is a developmental disorder characterized by deficits in social communication and repetitive and stereotyped interests and behaviors [1]. Autism is among the most enigmatic disorders of child development, with a dramatic increase in prevalence from 1 in 88 children in 2008 to 1 in 68 children in 2010 [2]. While the global burden of ASD is currently unknown, in the United States, the annual societal cost of the condition was recently predicted to be $126 billion and $34 billion in the UK [3]. This escalation and economic burden identify individuals with ASD as one of the highest priority populations for clinical research and treatment development.

Currently, one of the most burdensome complaints among parents of children with autism is disrupted sleep, with more than 40–80% of children experiencing sleep problems, compared with 25–40% in typically developing children (TYP) [4, 5]. In a developing child, sleep serves multiple functions, including energy conservation, brain growth, memory consolidation, and cognition [6]. Given the importance of sleep in daily functioning, the consequence of disrupted sleep in individuals with ASD is potentially serious. Recent research has shown that insufficient sleep exacerbates the severity of core ASD symptoms (e.g., repetitive behaviors, social and communication difficulties) [7, 8], as well as other maladaptive behaviors (e.g., self–injury, tantrums, and aggression) [9, 10]. To date, however, the relationship between sleep profiles and behavioral problems in individuals with ASD is limited. Current sleep treatments fail to target the specific nature of deficits in individuals with low-functioning autism. In this paper, we emphasize that the identification sleep profiles in children with low-functioning autism are necessary to identify targeted interventions, particularly for challenging behaviors in this disorder. This review concludes with methodological considerations and offers suggestions for future research designed to more clearly understand disrupted sleep so as to provide targeted treatments in this population.

### Low-functioning autism

ASD is characterized by notable phenotypic heterogeneity, which is often viewed as an obstacle to the study of etiology, diagnosis, treatment, and prognosis [11]. The degree of impairment among individuals with ASD is variable, thereby requiring the distinction between individuals with low-functioning autism and high-functioning autism, defined as those which have an intellectual quotient that is below average (<70) and above average (≥70), respectively [1]. What the current DSM-V fails to capture is that individuals with low-functioning autism experience significantly graver impairments than those experienced by their higher functioning counterparts [12]. In addition to displaying core symptoms of ASD, many children with low-functioning autism may exhibit serious behavioral disturbances such as tantrums, aggression, environmental destruction, socially inappropriate behavior, and self-injurious behavior [13]. Therefore, a child with low-functioning autism is likely to have a much more complex diagnostic picture, including a greater severity of ASD symptoms and associated co-morbidities and often require life-long extensive support. To date, these groups of individuals have not received comparable attention compared to individuals with high-functioning autism. This paper argues that these groups of individuals should be the focus of future research as they are most in need of treatment.

### Sleep difficulties in autism spectrum disorder

ASD is frequently accompanied by co-morbid disorders and associated problems, one of which is sleep disruption [14, 15]. One of the most burdensome and profound complaints among parents and caregivers of children with ASD is poor sleep. Research suggests that about 40–80% of individuals with an ASD experience a sleep problem, and the risk appears to be unrelated to the severity of cognitive impairment [16]. Other researchers have shown that individuals with low-functioning autism have a higher predisposition to chronic sleep-wake cycle disturbances when compared to higher-functioning individuals, given the degree and severity of their cognitive impairment [17]. This paper argues that understanding, identifying, and treating sleep disorders in low-functioning autism may impact favorably on associated conditions and daytime behavior and therefore improve the quality of life in this population.

### Heterogeneity of sleep difficulties in ASD

Since ASD is considered to be a multifaceted disorder reflected in different symptom profiles across individuals, it is not surprising that a multitude of sleep problems are prevalent in this population. Moreover, the variability of sleep profiles in ASD is suggestive of the mixed phenotypic profiles of ASD samples. Among children with ASD, the most common sleep issues are prolonged sleep latency, decreased sleep efficiency, reduced total sleep time, increased waking after sleep-onset, bedtime resistance, and daytime sleepiness; see [18] for a review. Accordingly, there does not appear to be one particular sleep problem that characterizes children with ASD, but many. These sleep difficulties appear to persist throughout the lifespan [19] and individuals with ASD who experience one sleep problem will often experience co-existing sleep problems [20]. Several of these sleep difficulties can be classified according to the International Classification of Sleep Disorders (ICSD-3) as primary sleep disorders (e.g., insomnia, parasomnia, and circadian rhythm sleep-wake disorders) [21]. In Table 1, the most common sleep problems in ASD have been reported against the ICSD-3 broad criteria for classifying sleep disorders in order to give a sense of the range and scope of sleep difficulties that are present in ASD. To date, most of the studies exploring sleep in ASD have focused on individuals with high-functioning autism, those individuals who have an ability to communicate and cooperate during actigraphy and polysomnography sleep studies [22]. Currently, there is an inconsistent understanding of the nature and prevalence of sleep difficulties in low-functioning autism. One study has suggested that the severity of sleep problems (such as sleep-onset delay and sleep duration) increases with the severity of autism symptoms (such as communication deficits) [8]. Another study has suggested that increased autism severity predicts an increased likelihood of sleep problems [23]; however, these links are still speculative, and sleep profiles in low-functioning autism are yet to be elucidated. To date, it is still unclear what specific sleep problems and symptom relationships are unique to individuals with low-functioning autism.

**Table 1:** **ICSD-3 Classification of sleep disorders in children with ASD including descriptions and evidence**

ICSD-3 Classification	Sleep profile	Study	Sleep measures	Significant findings in ASD population
Insomnia	Persistent difficulty with sleep initiation, maintenance, duration, consolidation, or quality. Includes bedtime resistance, frequent night awakenings, and/or an inability to sleep independently	Wiggs *et al.* [24]	Actigraphy and SQ	Increased sleep latency, night awakenings, and poor sleep efficiency
		Malow *et al.* [25]	PSG and CSHQ	Poorer sleep efficiency, longer sleep latency, and frequent night awakenings (up to 2–3 h)
		Goodlin-Jones *et al.* [26]	Actigraphy and SD	Less total sleep time (TST) compared to TYP children or those with a DD
		Krakowiak *et al.* [27]	SQ	Higher sleep-onset factor scores and night awakenings compared to typical children
		Souders *et al.* [28]	CSHQ, SD, and actigraphy	Behavioral insomnia evident in 66% of children with ASD compared to 45.9% in controls
		Anders *et al.* [29]	Actigraphy and SD	ASD children aged 2–5 years slept less per 24-h period on average compared to controls
		Giannotti *et al*. [30]	PSG and CSHQ	Children with regressive ASD (*n* = 18) had greater bedtime resistance, sleep-onset latency, and less TST than controls
		Sivertsen *et al.* [31]	Parent report	Prevalence of chronic insomnia was ten times higher in children with ASD symptoms compared to controls
		Baker *et al.* [32]	Actigraphy and SD	Adolescents with ASD were three times more likely to have symptoms of insomnia than their TYP peers
Parasomnias	Undesirable physical experiences which occur within sleep or during arousal from sleep. Includes nightmares, wake screaming, complex movements, dreams, and automatic nervous system activity	Hering *et al.* [33]	Actigraphy and SQ	54% of children with ASD had multiple and early night arousals
		Doo *et al.* [34]	SQ, CSHQ, and actigraphy	All reported evidence of higher rates of parasomnias in children with ASD compared to comparison groups
		Schreck *et al.* [35]		
		Liu *et al.* [20]		
		Goldman *et al*. [36]	CSHQ	Younger children with ASD had more parasomnias than older children
Circadian rhythm sleep-wake disorders	Alterations of the circadian time-keeping system, its entrainment mechanisms, or misalignment of the endogenous circadian rhythm and the external environment. Manifests in difficulty initiating and maintaining sleep	Giannotti *et al.* [37]	PSG and CSHQ	More than 10% of children with ASD were found to have sleep problems that varied by season due to fluctuations in light/dark cycles
		Tordjman *et al.* [38]	Measures of melatonin	Elevated daytime and lower nocturnal melatonin in individuals with ASD compared with controls
		Hayashi [39]	SD, CSHQ, and PEQ	“Free-running” sleep (not entrained to 24-h), sleep-onset delay, and early morning awakening in children with ASD
		Segawa [40]		

*CSHQ* Child Sleep Habit Questionnaire, *DD* developmental disability, *TYP* typical development, *PEQ* Parenting Events Questionnaire, *PSG* polysomnography, *SD* sleep diary, *SQ* Sleep Questionnaire.

### The complex etiology of sleep disturbances in individuals with ASD

Although highly prevalent and persistent, the etiology of sleep problems in children with ASD remains uncertain [41]. Several theories have been put forward to suggest that sleep disruption may be a direct result of either i) the ASD condition or ii) other associated co-morbidities. Research has suggested that the underlying neurophysiology and neurochemistry may predispose individuals with ASD to have chronic sleep-wake disturbances. The development of circadian rhythms, which is established within 12–16 weeks of birth, requires the perception of the environmental time cues (“zeitgebers”) to permit appropriate entrainment with the 24-h day (i.e., the synchronization of the internal biological clock to external time cues). The most powerful time cue is the 24-h light/dark cycle, but non-photic time cues such as timing of meals and social contacts can also have influence. Where brain damage or maldevelopment has occurred such as in ASD, these entrainment pathways may be impaired [19]. Children with low-functioning autism are subject to many variables that can potentially affect circadian entrainment, including decreased sensitivity to social cues, and possible misalignment between circadian phase and imposed light/dark cycles due to variations in light sensitivity [42]. There is also evidence of biological abnormalities in the timing of melatonin secretion (a neurohormone which regulates the sleep-wake cycle). Studies have shown elevated daytime melatonin and significantly less nocturnal melatonin in individuals with ASD compared to controls [43]. Other studies have found variability in melatonin production with some individuals having normal melatonin profiles, suggesting that there is a subgroup of individuals with ASD that may have a dysregulation in their circadian rhythms [43, 44]. It is also important to note that melatonin abnormalities have been found in several other disorders with intellectual disability [45, 46], raising the issue of non-specificity of the melatonin findings in ASD [38]. Nevertheless, early speculations suggest that the influence on melatonin and altered rhythms in a subset of children with ASD may lead to differences in sleep schedules and result in perceived problematic behavior around bedtime and morning routines.

Other theories have been put forward to suggest that sleep disruption may be a secondary condition influenced by other co-occurring medical and psychiatric conditions that are present in ASD. Gastrointestinal disorders (GI) are common in ASD children with more than 50% of children experiencing constipation or diarrhea, often resulting in induced toilet awakenings throughout the night [47]. Seizures and epilepsy are also common in children with low-functioning autism (6–60%), and research has shown that sleep deprivation can often facilitate seizures, and conversely, seizures adversely affect sleep architecture [48]. Children with ASD are also 25–70% more likely to have co-morbid psychiatric conditions such as anxiety and attention-deficit/hyperactivity disorder (ADHD), displaying symptoms of inattention and high levels of arousal such as hyperactivity [49]. These conditions influence pre-sleep arousal, significantly delayed sleep onset, and over time may be linked to the development of insomnia [50]. Medications known to treat medical and behavioral conditions, such as antipsychotics and serotonin reuptake inhibitors (SSRIs) may also disrupt the sleep-wake cycle in ASD [18]. Understanding the nature of sleep disturbances in ASD is a complex dynamic process whereby there is a multi-directional relationship between sleep and certain factors. Further exploration of such relationships may elucidate mechanisms, which may in turn suggest effective treatment strategies to reduce sleep symptoms in individuals with low-functioning autism.

### Treating sleep disruptions in children with ASD

There is increasing evidence of severe sleep problems in children with autism, although little research exists for evidence-based sleep treatments within this population [51]. Sleep disorders in ASD often remain untreated and ignored as other behavioral difficulties tend to take precedence [52]. Some research supports the efficacy of melatonin in decreasing sleep-onset latency and increasing total sleep time when administered close to bedtime [53, 54]. In contrast, studies have suggested that melatonin is only effective for children with ASD who have difficulties with sleep latency, as it is known to increase night awakenings and disrupt sleep maintenance [55]. Additionally, the effectiveness of melatonin is influenced by the type of sleep disturbance, environmental factors, and other associated medical conditions [56]. After excluding biological factors, parent-based education and behavioral interventions are the first line of treatment for sleep disruption in ASD [57]. Behavioral interventions such as sleep hygiene approaches which focus on changing the environment in order to promote regular sleep-wake cycle have been shown to be effective interventions in improving sleep onset and maintenance in ASD [58]. The basic principles of sleep hygiene include selecting an appropriate bedtime and set routine, minimizing television watching, and reducing emotional and behavioral stimulation at night [16]. This behavioral treatment approach is optimal for individuals with low-functioning autism who on average have minimal or no verbal skills. Currently, however, the efficacy of behavioral treatment approaches is based on small studies and lack objective sleep measures and has been performed with the inclusion of children having a variety of diagnoses not limited to ASD [59]. Light therapy is effective in advancing or delaying the sleep phase in patients with circadian sleep disorders and can be considered for children with an ASD who present with circadian dysfunction [16]; however, there is limited research available with light therapy with individuals with low-functioning autism. Given the associations between inadequate sleep, intensified daytime problem behaviors, and parental stress in ASD, there is a strong need to develop effective sleep interventions adapted to a child’s cognitive and developmental level.

### The relationship between poor sleep and challenging behaviors in ASD

In typical development, sleep disruption is associated with emotional and behavioral problems such as internalizing and externalizing symptoms [60]. Moreover, a growing body of evidence shows that childhood sleep disturbances may widely impact children’s health, behavior, attention, cognition, and school performance [61]. Given the nature of autism and its associated challenging behaviors, the effects of sleep disruption in this disorder are potentially serious. Sleep problems have been found to exacerbate ASD symptoms. Fewer hours of sleep have been shown to correlate with and predict greater ASD severity such as social skill deficits [41], communication impairments, higher rates of stereotypic behaviors, and stricter adherence to non-functional routines [62]. In addition to exacerbating ASD symptoms, sleep difficulties have been shown to be associated with increased rates of over-activity, disruption, non-compliance, aggression, irritability, and affective problems, which are all problems that could significantly interfere with daytime functioning in ASD [19, 25, 62, 63]. Table 2 summarizes studies conducted to date exploring the relationship between sleep and challenging behaviors, ordered by date of publication, and summarized by their method, significant findings, and effect sizes. Despite the research exploring the relationship between sleep difficulties and challenging behavior in ASD, the influence of sleep problems in children with low-functioning autism has been neglected. Moreover, limited research has been conducted on the bi-directional relationship between sleep and behavior in these individuals.

**Table 2:** **Studies exploring the relationship between sleep and challenging behaviors in ASD**

Study	Type of study	Participants	Measurements	Significant findings	Effect sizes ( ***r***) ^1^
Schreck *et al.* [62]	Cross sectional	55 parents of children with mixed ASD functioning aged 5–12 years	GARS, BEDS, and PSQ	Fewer hours of sleep per night predicted ASD severity score, social skill deficit, and stereotypic behavior	0.33–0.34^b^
Liu *et al.* [20]	Cross sectional	27 children with ASD symptoms, and 32 with other DD (*M* = 8.8)	ADOS, CHSQ, and PSQ	Hypersensitivity to stimuli, younger age, co-sleeping, medication, epilepsy, history of sleep problems, and ADHD was associated with sleep problems in individuals with ASD	0.09^a^–0.31^b^
DeVincent *et al*. [64]	Cross sectional	Parents of children with PDD (*n* = 112) and TYP (*n* = 497) aged 3–5	Early childhood inventory-4 and PSQ	PDD children with sleep problems had higher rates of ADHD, oppositional behavior, and psychiatric symptoms compared to children without sleep problems	0.22–0.26^a^
Goodlin-Jones *et al.* [65]	Cross sectional	68 HFASD children, matched to 57 with DD, 69 TYP, aged 24–69 months	Actigraphy, MELC, VABC, ADOS, ADIR, MSEL, SD, CSHQ, ESS, and CBCL	Controlling for diagnosis and age, night-time sleep problems determined by parent report were significantly associated with decrements in daytime behavior	0.30–0.43^b^
Mayes *et al.* [66]	Cross sectional	Parents of 477 children with a range of ASD diagnoses (aged 1–15)	CARS, PBS, PSQ, WISC, WPPSI, and GDS	Sleep problems increased with severity of ASD symptoms. Oppositional behavior, aggression, ADHD, and mood variability predicted sleep disturbance in ASD	0.59^c^
Goldman *et al.* [67]	Cross sectional	42 mixed ASD samples and 16 TYP children aged 4–10 years	PCQ, CSHQ, RBS-R, CBCL, PSG, and Actigraphy	Poor sleepers with ASD had more ADHD symptoms and more restricted and repetitive behaviors (RRBs) than good sleepers. Sleep fragmentation was correlated with more RRBs	0.48^b^–0.69^c^
Moon *et al.* [68]	Case study	3 children (aged 8–9 years) diagnosed with an ASD	Actigraphy, SD, CSHQ, CBCL, and PSQ	Daytime behavior improved for 2/3 ASD patients following an intensive sleep treatment	*x*
Rzepecka *et al.* [69]	Cross sectional	187 parents with child aged 5–18 years with an ID and/or ASD from Scotland	ADOS, CSHQ, SCAS-P, and ABC	Sleep problems were the highest predictor of challenging behaviors in ASD	0.62^c^
Henderson *et al.* [9]	Cross sectional	Parents of children aged 6–12 years with ASD, Asperger’s (*n* = 58), and non-ASD (*n* = 57)	CSBQ, CRQ, BRQ, CSHS, CSWS, and CBCL	In the ASD group, poor sleep quality and hygiene were related to higher levels of externalizing behaviors	0.60^c^
Goldman *et al.* [10]	Cross sectional	Parents of 1,784 children, ages 2–18 with high-functioning autism (USA)	ADOS, CHSQ, and PCQ	Poor sleepers had a higher percentage of behavioral problems on all PCQ scales (e.g., aggression, RRBs, stereotypy, and hyperactivity) than good sleepers	0.11^a^–0.34^b^
Sikora *et al*. [70]	Cross sectional	Parents of 1,193 children with mixed ASD diagnosis aged 4–10 years (USA)	CSHQ, VABS, and MSEL	Moderate-severe sleep problems in ASD resulted in higher daytime externalizing behavior and poorer adaptive skills than those with ASD with no sleep problems	*x*
Anders *et al.* [71]	Cross sectional	Parents of children with an ID (*n* = 57), ASD (*n* = 68), and TYP (*n* = 69), aged 24–66 months	ADOS, ADIR, MSEL, CBCL, actigraphy, CSHQ, and WISC	Parent-reported sleep problem but not actigraphy recordings were associated with more core behavior problems in ASD	0.12^a^–0.39^b^
Tudor *et al.* [8]	Cross sectional	Parents of 109 children with a diagnosis of ASD (aged 3–18 years)	CSHQ, GARS, and PECS board	Sleep-onset delay and duration was positively correlated with ASD severity and symptoms and was the strongest predictor of communication deficits and stereotypic behavior	0.34^b^–0.51^c^
Park *et al.* [7]	Cross sectional	Parents of 166 ASD children and 111 unaffected siblings aged 4–15 years from Korea	ADIR, ADOS, CSHQ, WISC, and K-CBCL	Communication abnormalities and RRBs were associated with increased risk of sleep problems in ASD. ASD individuals had higher, internalizing, and externalizing problems compared to their unaffected siblings	0.31–0.43^b^
Taylor *et al.* [72]	Cross sectional	Parents of children with an ASD (*n* = 335) aged 1–10 years (*M* = 5.5)	BEDS, WISC, WPPSI, MSEL, SIB-R, and VABS	Children who slept fewer hours per night had lower IQ, verbal skills, adaptive functioning, socialization, and communication skills	0.40–0.44^b^
Holloway *et al.* [73]	Cross sectional	1,583 ASD children from Autism Treatment Network aged 2–17 years	CSHQ, VABS, MSEL, Stanford Binet, CBCL, ADOS, and SSP	Anxiety, ASD severity, sensory sensitivity, and GI issues all predicted sleep disturbance. IQ positively predicted sleep disturbance	0.17^a^–0.44^b^
Schwichtenberg *et al*. [74]	Cross sectional	ASD siblings (*n* = 104) and families with no history of ASD (*n* = 76)	MSEL, ADOS, CBCL, and PCQ	For both groups, sleep problems were associated with elevated behavior problems (e.g., reactivity, anxiety, somatic complaints, withdrawal, inattention, and aggression)	0.16–0.21^a^
Mannion *et al.* [47]	Cross sectional	Parents of 89 children and adolescents (aged 3–16) with mixed ASD subtypes in Ireland	ASD-CC, GSI, and CSHQ	Avoidant behavior, under-eating, and GI symptoms predicted sleep problems in individuals with an ASD	0.46^b^–0.50^c^
May *et al.* [75]	Longitudinal	Gender-matched children with high-functioning autism (*n* = 46) and TYP (*n* = 38) aged 7–12 years from Melbourne (Australia)	Conner’s third edition, SCAS, and CSHQ	The ASD group had more sleep disturbance than the TYP group. Sleep disturbance decreased over the year in children with ASD, and this was associated with improved social ability	0.41^b^–0.69^c^
Richdale *et al.* [50]	Cross sectional	27 adolescents with high-functioning autism (aged 15–16) and 27 matched TYP controls	SD, actigraphy, CSRQ, CED-S, DASS-21, and SAAQ	Sleep variables significantly accounted for 57% of the variance of daytime functioning symptoms of insufficient sleep in the high-functioning ASD group	0.75^c^
Adams *et al.* [23]	Cross sectional	548 children and adolescents (2–18 years), with ASD symptoms	ASD-CC	Individuals with severe sleep problems had higher levels of total challenging behaviors than those with mild sleep problems	−0.47^b^

*ABC* Aberrant Behavior Checklist, *ADIR* Autism Diagnostic Interview (revised), *ADOS* Autism Diagnostic Observation Schedule, *ASD-CC* autism spectrum disorder co-morbid for children, *ASD* autism spectrum disorders, *BEDS* bedtime evaluation of disorders of sleep, *BRQ* Bedtime Routines Questionnaire, *CARS* Checklist for Autism Spectrum Disorders, *CBCL* Child Behavior Checklist, *CES-D* Centre for Epidemiological Studies Depression Scale, *CRQ* Child Routine Questionnaire, *CSBQ* Children’s Social Behavior Questionnaire, *CSHQ* Child Sleep Habit Questionnaire, *CSHS* Children’s Sleep Hygiene Scale, *CSRQ* Chronic Sleep Reduction Questionnaire, *CSWS* Children’s Sleep-Wake Scale, *DASS-21* Depression, Anxiety, and Stress Scale, *DD* Developmental disability, *ESS* Epworth Sleepiness Scale, *GARS* Gilliam Autism Rating Scale, *GDS* Gordon Diagnostic System, *GI* Gastrointestinal disorder, *GSI* Gastrointestinal Symptoms Inventory, *HFASD* High-functioning autism spectrum disorder, *ID* Intellectual disability, *IQ* Intellectual quotient, *K-CBCL* Korean Version of Child Behavior Checklist, *MELC* Mullen Early Learning Composite, *MSEL* Mullen Scales of Early learning, *PEQ* Parenting Events Questionnaire, *PBS* Pediatric Behavior Scale, *PCQ* Parental Concerns Questionnaire, *PDD* Pervasive developmental disorder, *PPVT* Peabody Picture Vocabulary Test-III, *PSG* Polysomnography, *PSQ* Parental Sleep Questionnaire, *RBS-R* Repetitive Behavior Scales-revised, *RRB* Repetitive and restricted behaviours, *SAAQ* Sleep Anticipatory Anxiety Questionnaire, *SCAS-P* Spence Anxiety Scale Parent Version, *SD* Sleep diary, *SIB-R* Scales of Independent Behavior-Revised, *SIB* self-injurious behavior, *SQ* Sleep Questionnaire, *SSP* Short Sensory Profile, *TYP* typical development, *VABC* Vineland Adaptive Behavior Checklist, *WISC* Wechsler Intelligence Scale for Children, *WPPSI* Wechsler Preschool and Primary School Scale of Intelligence, *x* insufficient information provided to calculate effect sizes.

^a^small (*r* ≥0.1), classification of effect size.

^b^medium (*r* > 0.30), classification of effect size.

^c^large (*r* > 0.5), classification of effect size.

### Future directions: the relationship of sleep and behavior in ASD

#### Researched areas

The current research highlights clear uni-directional relationships between sleep and behavior in individuals with an ASD. It is well researched that sleep problems worsen ASD symptomatology across most core domains, as well as exacerbate pre-existing behavioral problems. These relationships have been fairly well investigated in cross-sectional studies using objective measures with individuals with mixed groups of ASD samples and individuals with high-functioning autism. Objective tools such as polysomnography (a tool that monitors physiological parameters during sleep such as electroencephalogram) and wrist actigraphy (a tool that uses an accelerometer to detect and record muscle activity) have been used successfully to validate relationships between poor sleep and daytime behaviors in a mixed sample of ASD children [51, 65, 71]. There is also modest evidence to suggest that holistic parent report measures such as the CSHQ is a superior single-item response measure which helps gage overall quality of ASD children’s sleep [76]. In light of knowledge about the severity of sleep disorders in ASD, there is evidence to suggest that sleep is amendable to treatment in certain populations with ASD. Studies have shown that parent-based education (behavioral therapy) improves sleep-onset delay in children with high-functioning autism [59] and that pharmacological treatments such as melatonin is an effective sleep treatment for children with autism [77]. Moreover, treating sleep in a subgroup of individuals with ASD has been shown to improve core ASD symptoms (e.g., communication and socialization impairments) as well as reduce the severity of challenging behaviors in ASD [38, 68]. Applied behavior analysis (ABA) treatment approach is known to be efficacious for the treatment of challenging behavior in a minority of children with ASD [62]; however, its outcomes are influenced by learning rate and cognitive performance. Given that sleep is implicated in behaviors that affect learning, such as compliance, irritability, hyperactivity, and aggression, there is now more evidence to suggest that sleep is a possible obstacle to ABA treatment success in ASD [62]. Given the bi-directional relationship between challenging behaviors and sleep disturbance in ASD, preliminary evidence suggests that treating sleep disturbances and challenging behavior in isolation may not lead to successful outcomes [50]. The foregoing relationship between sleep problems for children with ASD and daytime inappropriate behavior suggests additional research is required to delineate direct connections among specific sleep problems and the specific daytime behavior patterns that may affect individuals with ASD.

#### Areas for further research

Although previous studies have identified clear relationships between poor sleep and challenging behaviors in ASD, as reviewed above, it is still unclear what specific sleep problems and symptom relationships are unique to individuals with low-functioning autism. As mentioned, current research has primarily focused on individuals on the higher functioning end of the autism spectrum, and individuals with low-functioning autism who potentially have the most severe sleep and behavioral deficits have been relatively ignored in the literature. Studying sleep in children with low-functioning autism presents with unique methodological challenges, namely subjective parent reports confer reporting bias and negative halo effects [78] and individuals have difficulty tolerating objective measures such as PSG and actigraphy tools due to sensory sensitivities and lack of cooperation [76]. Given that the National Sleep Foundation [79] identifies children with ASD as one of the highest priority populations for sleep research, there is a need for more accurate, objective, non-invasive measures of sleep, as well as data from children with low-functioning autism in order to better characterize the quality and quantity of sleep in this population.

Another key limitation of the research to date is that very few studies examine behavioral problems and sleep disturbances in ASD longitudinally, with most studies being cross-sectional. Cross-sectional studies only capture an ASD profile at one specific age presentation, and most studies have combined both children and adolescents in their samples. Little is known about how sleep changes over time in ASD and what factors might be associated with this change, for example, age and stages of development. In ASD, one study found no relation between sleep difficulties and developmental stage (i.e., childhood, adolescents, or adulthood) [66], whereas other studies, albeit cross sectional, have found a decline in sleep difficulties with age similar to typical development [36, 37]. The severity of ASD symptoms and behavioral disturbance have been known to wax and wane across development, with some behaviors improving with age [80]. Given that different behavioral profiles occur at particular age ranges and developmental age often does not match chronological age in ASD, there is a need to study relationships through longitudinal designs. Only one study to date has compared the relationship between sleep disturbances and behavior longitudinally, in high-functioning autism and in typically developing controls [75]. Thus, to uncover core ASD phenotypes and link these to sleep profiles, more longitudinal studies are required to trace sleep trajectories in this population to understand what unique variables might influence change. For example, sleep difficulties in ASD may vary according to medication use, environment factors such as seasonal changes, or co-morbidities such as epilepsy or GI issues. It is difficult to determine whether co-occurring conditions cause the behavior problems, maintain existing problems, or exacerbate problems already present in ASD. Studies need to be done to address this poignant question.

Lastly, treatment guidelines to help manage challenging behaviors in individuals with low-functioning autism often fail to mention sleep at all, or they present a very limited account. Identifying and providing treatment for sleep problems in ASD is imperative for improving sleep, as well as for encouraging more positive prognoses by improving daytime behavior and family functioning in this population. One specific proposal is for researchers to identify factors that result in an ASD phenotype and then design targeted therapeutic interventions to reverse or ameliorate specific deficits. For example, exposure to highly arousing stimuli before bed may increase pre-sleep arousal and sleep-onset latency and result in an increase in self regulatory behaviors (such as self-injurious behaviors) the following day in children with low-functioning autism. Heightened light sensitivity from exposure to blue-enriched light from computer and/or tablet screens might also be linked to circadian timing and melatonin issues, increasing sleep-wake circadian rhythm abnormalities in this population. Sound sensitivity inherent within ASD may be linked to lower waking thresholds, sleep fragmentation, and so on. In this paper, it is proposed that profiling ASD children based on the nature of their sleep disruption might help understand symptom and behavioral profiles (or vice versa) and therefore lead to better-targeted interventions.

## Conclusions

Although there is reason to believe that serious sleep problems are common in children with ASD and that poor sleep exacerbates problematic daytime behavior, these conclusions are still premature and require further investigation. Gaining more specific insight into the individual nature of sleep difficulties in ASD opens up a novel avenue for designing interventions, as sleep is an area with a potential for remediation. Since sleep is a central mechanism for adaptive functioning (e.g., learning, memory, neuroplasticity), it is highly plausible that sleep deficits play a leading role in the symptoms seen in ASD including the exacerbation of challenging behaviors. To date, however, studies have failed to provide conclusive evidence about the relationship between sleep and behaviors seen in low-functioning individuals (of all ages) with autism. This review highlights the value of defining sleep profiles for children with ASD and integrating different aspects of their symptom profile to their sleep deficits (and vice versa). In turn, this knowledge will result in novel therapeutic targets and interventions that will hopefully improve long-term outcomes of nearly 1 in 68 individuals affected by this pervasive, developmental disorder.

## Abbreviations

ABC: Aberrant Behavior Checklist
ADHD: Attention deficit hyperactivity disorder
ADIR: Autism Diagnostic Interview (revised)
ADOS: Autism Diagnostic Observation Schedule
ASD-CC: Autism spectrum disorder co-morbid for children
ASD: Autism spectrum disorders
BEDS: Bedtime evaluation of disorders of sleep
BRQ: Bedtime Routines Questionnaire
CARS: Checklist for Autism Spectrum Disorders
CBCL: Child Behavior Checklist
CRQ: Child Routine Questionnaire
CSBQ: Children’s Social Behavior Questionnaire
CSHQ: Child Sleep Habit Questionnaire
CSHS: Children’s Sleep Hygiene Scale
CSWS: Children’s Sleep-Wake Scale
DD: Developmental disability
ESS: Epworth Sleepiness Scale
GARS: Gilliam Autism Rating Scale
GDS: Gordon Diagnostic System
GSI: Gastrointestinal symptoms inventory
HFASD: High-functioning autism spectrum disorder
ID: Intellectual disability
IQ: Intellectual quotient
K-CBCL: Korean Version of Child Behavior Checklist
LFASD: Low-functioning autism
MELC: Mullen Early Learning Composite
MSEL: Mullen Scales of Early Learning
PEQ: Parenting Events Questionnaire
PBS: Pediatric Behavior Scale
PCQ: Parental Concerns Questionnaire
PDD: Pervasive developmental disorder
PPVT: Peabody Picture Vocabulary Test-III
PSG: Polysomnography
PSQ: Parental Sleep Questionnaire
RBS-R: Repetitive Behavior Scales-Revised
RRB: Repetitive and restricted behaviours
SCAS-P: Spence Anxiety Scale Parent Version
SD: Sleep diary
SIB-R: Scales of Independent Behavior-Revised
SIB: Self-injurious behavior
SQ: Sleep questionnaire
SSP: Short Sensory Profile
TYP: Typical development
VABC: Vineland Aberrant Behavior Checklist
WISC: Wechsler Intelligence Scale for Children
WPPSI: Wechsler Preschool and Primary School Scale of Intelligence.
